# Impact of ISTA and FISTA iterative optimization algorithms on electrical impedance tomography image reconstruction

**DOI:** 10.2478/joeb-2025-0003

**Published:** 2025-03-11

**Authors:** Quoc Tuan Nguyen Diep, Hoang Nhut Huynh, Thanh Ven Huynh, Minh Quan Cao Dinh, Anh Tu Tran, Trung Nghia Tran

**Affiliations:** Laboratory of Laser Technology, Ho Chi Minh City University of Technology (HCMUT), Ho Chi Minh City 72409, Vietnam; Vietnam National University Ho Chi Minh City, Linh Trung Ward, Thu Duc, Ho Chi Minh City 71308, Vietnam

**Keywords:** Electrical Impedance Tomography, iterative method, ISTA, FISTA, image reconstruction

## Abstract

Electrical Impedance Tomography (EIT) is a non-invasive method for imaging conductivity distributions within a target area. The inverse problem associated with EIT is nonlinear and ill-posed, leading to low spatial resolution reconstructions. Iterative algorithms are widely employed to address complex inverse problems. However, current iterative methods have notable limitations, such as the arbitrary and subjective selection of initial parameters, lengthy computational times due to numerous iterations, and the generation of reconstructions that suffer from shape blurring and a lack of higher-order detail. To address these issues, this study investigates the impact of using ISTA and FISTA iterative algorithms on the image reconstruction process in EIT. It focuses on enhancing the convergence and accuracy of EIT image reconstruction by evaluating the effectiveness of these optimization algorithms when applied to regularized inverse problems, using standard regularization techniques. ISTA and FISTA were compared to the NOSER and Newton-Raphson methods and validated through simulation and experimental results. The results show that ISTA and FISTA achieve better visualization and faster convergence than conventional methods in terms of computational efficiency when solving regularized problems, achieving conductivity reconstructions with an accuracy of above 80%. The paper concludes that while ISTA and FISTA significantly enhance EIT image reconstruction performance, the quality of the reconstructed images heavily depends on the choice of regularization methods and parameters, which are crucial to the reconstruction process.

## Introduction

Electrical Impedance Tomography (EIT) has become an increasingly important technology, particularly in the fields of medical diagnostics, environmental monitoring, and industrial applications [1, 2, 3, 4, 5, 6, 7]. EIT enables visualization of the distribution of electrical properties within a specific region without the need for invasive techniques [8, 9, 10, 11, 12]. However, EIT has limitations, particularly in terms of spatial resolution, and requires precise knowledge of the experimental parameters, such as the exact position of the electrodes [13, 14, 15, 16]. This knowledge gap affects the robustness of the reconstruction process [17, 18, 19]. Previous research has made numerous contributions to improving image reconstruction accuracy and robustness, primarily by addressing modeling errors [13, 18, 20]. Regularization methods are crucial for enhancing the quality of reconstructed images by incorporating prior information into mathematical models [21, 22, 23].

These studies highlight the primary objective of achieving high precision for specific use cases in clinical settings, where managing experimental variables is challenging. Standard EIT systems often lack robustness against modeling errors, which become more pronounced when assessing pulmonary artery volume and pressure in electrode belt displacement or detachment [24]. These issues underline the importance of addressing electrode placement inaccuracies and other experimental variables [14]. Comprehensive reviews have focused on the latest developments in EIT methodologies and algorithms [20, 25].

Regularization methods play a crucial role in stabilizing the solutions of inverse problems by incorporating prior information into the mathematical models used for image reconstruction. Traditional regularization techniques, such as Total Variation (TV), impose constraints on the image or its gradients to control overfitting and enhance stability [26]. These methods have been widely studied and applied to improve the robustness and accuracy of the EIT image reconstruction.

Previous studies have introduced various regularization techniques to enhance the reliability of reconstructed images. For instance, Tikhonov regularization imposes linear constraints on an image or its gradient to mitigate overfitting and improve stability [27, 28]. The Gauss-Newton method combines Newton and Gauss techniques to iteratively update a linear loss function derived from the difference between actual and reconstructed data, addressing the issue of nonlinear inverses [29, 30]. Laplace regularization uses the Laplace transform to reduce noise and enhance the resolution, resulting in smoother and more accurate reconstructions [31]. The Newton’s one-step error re-constructor (NOSER) method focuses on minimizing errors at each step of the reconstruction process, thereby improving stability and reliability [32, 33]. TV regularization emphasizes minimizing image gradients, promoting high-definition and low-noise image reproduction by focusing on object boundaries [26].

Iterative methods commonly used in image reconstruction include the Newton-Raphson method, NOSER method, Iterative Shrinkage-Thresholding Algorithm (ISTA), and Fast Iterative Shrinkage-Thresholding Algorithm (FISTA) [34, 35]. The Newton-Raphson method approximates the objective function with a quadratic function to locate its extremum, providing an estimate of the minimum of the function [36]. This method is computationally intensive because it requires the Hessian matrix to be calculated in each iteration, particularly with many variables. The NOSER method simplifies the Newton-Raphson method to its first step, setting an initial conductivity to approximate the conductivity distribution of the field [34]. However, this approach only provides information on the location and size of the medium, but not its shape. ISTA and FISTA have gained attention for their efficiency in solving regularized inverse problems [37, 38]. It is important to note that ISTA and FISTA are not regularization methods but optimization tools used to solve problems that include regularization terms. These algorithms are effective for problems involving L1-norm regularization, which promotes sparsity in the solutions.

However, current iterative methods have notable limitations, such as the arbitrary and subjective selection of initial parameters, the lengthy computational time due to numerous iterations, and the generation of reconstructions that suffer from shape blurring and a lack of higher-order detail. To address these challenges, this study evaluates the impact of ISTA and FISTA on EIT image reconstruction when applied to issues regularized by methods such as total variation (TV). By comparing these optimization algorithms, we aim to understand their effectiveness in enhancing the quality and robustness of reconstructed images. This study also explored the influence of different regularization parameters and priors on the performance of these algorithms. By distinguishing between optimization algorithms and regularization methods and systematically evaluating their combined effects on EIT image reconstruction, this study seeks to advance more accurate and reliable EIT imaging solutions for clinical and industrial applications.

## Method

### Inverse Problem

In EIT, the reconstruction of conductivity maps within a medium is achieved by injecting alternating current (I) through strategically placed electrodes and measuring the resulting voltage (V) at the surface [3]. This process involves employing the Finite Element Method (FEM) to model the medium, account for electrode placements, and solve the associated inverse problem using various regularization techniques [39]. A commonly adopted practical approach is the adjacent measurement strategy, in which voltage measurements are not performed at the current injection electrodes. This strategy reduces redundant data by systematically rotating the current injection and measurement points, thereby improving the data acquisition efficiency.

EIT image reconstruction involves solving both forward and inverse problems simultaneously. The complexity of the forward problem is influenced by the FEM mesh and the number of electrodes [14]. However, the inverse problem requires the construction of a numerical model that discretizes the domain into fine elements to approximate the conductivity distribution. A widely used approach to solving the inverse problem is time-difference imaging, which aims to find a stable resistivity distribution (*ρ*) that minimizes the difference between the measured voltages (V) and the model’s predicted voltages, *V* (*ρ*) as shown in (1).

(1) $$\min{\left\|V-V(\rho )\right\|}^{2}$$

where V(*ρ*) represents the baseline voltage measured under homogeneous conditions or at a distinct temporal point.

Because the inverse problem in EIT is inherently nonlinear and ill-posed, estimating the impedance distribution requires careful regularization to obtain a stable and meaningful solution. Regularization incorporates prior information to constrain the solution, as shown in (2).

(2) $$\min{\left\|V-V(\rho )\right\|}^{2}+\alpha {\left\|L\rho \right\|}^{2}$$

where *α* is the regularization parameter, and *L* is the regularization operator, which can enforce smoothness or other constraints on the solution. Regularization enhances the agreement between the estimated and measured voltages, and its effectiveness is strongly dependent on the chosen method. Iterative algorithms, such as ISTA and FISTA, were employed to solve the regularized cost function. By optimally adjusting *α* and iteratively refining the solution, these methods play a critical role in achieving accurate and reliable IT-image reconstruction.

### ISTA and FISTA Iterative Optimization

ISTA and FISTA are iterative optimization algorithms designed to solve regularized inverse problems. Although these algorithms are not regularization methods, they efficiently compute solutions to problems formulated with regularization terms. ISTA and FISTA are particularly effective for L1-norm regularization, such as sparse signal reconstruction. These algorithms iteratively refine the solution, progressively reducing the error between the measured and reconstructed data, guided by the chosen regularization method [40, 41, 35].

Proximal operators and the proximal gradient method are fundamental tools in optimization, for solving problems involving non-smooth functions [42, 43]. The proximal operator for a given function *f*, denoted as prox_λ*f*_(*v*), is obtained by determining the argument *x* that minimizes the expression in (3).

(3) $$f(x)+\frac{1}{2\lambda }{\left\|x-v\right\|}^{2}$$

where *λ* > 0 is the parameter. A notable property of this operator is that a point x^*^ minimizes the function *f* if and only if it is a fixed point of prox_λ*f*_ [44], which can be expressed as in (4).

(4) $$x^{*}={\text{prox}}_{\lambda f}\left(x^{*}\right)$$

This result is particularly useful when deriving the proximal operator for the L1-norm, *f* (*x*) = ∥*x*∥_1_. The minimum function can be determined as shown in (5).

(5) $$\phi (x,v)={\left\|x\right\|}_{1}+\frac{1}{2\lambda }{\left\|x-v\right\|}^{2}$$

Differentiating this function element-wise for *x* and setting the result to zero yields the result shown in (6).

(6) $$\frac{\partial \phi }{\partial x_{i}}=\text{sign}(x_{i})+\lambda (x_{i}-v_{i})=0$$

By solving for *x_i_*, we obtain the result as shown in (7).

(7) $$x_{i}=\left\{\begin{array}{ll} v_{i}-\lambda & \text{if}\;v_{i}<-\lambda \\ 0 & \text{if}\;-\lambda \leq v_{i}\leq \lambda \\ v_{i}+\lambda & \text{if}\;\;v_{i}>\lambda \end{array}\right.$$

The soft-thresholding operator, denoted by *S*_λ_(*u*), can be more succinctly expressed as shown in (8).

(8) $$S_{\lambda }(u)=\text{sign}(u)\max\left(\left|u\right|-\lambda ,0\right)$$

A mapping is considered Lipschitz continuous if a constant *L* exists, satisfying the condition [45] as shown in (9).

(9) $${\left\|f(x)-f(y)\right\|}_{2}\leq L\left\|x-y\right\|$$

If the Lipschitz constant *L* is less than 1 (*L* < 1), the mapping is termed as contractive. Conceptually, a contractive map reduces the distance between points upon application, potentially leading to convergence to a fixed point through an iterative application. The Lipschitz constant *L* is particularly significant in discussions related to iterative soft-thresholding algorithms (ISTA), as it allows the verification of a function’s contractivity by adjusting the scale of the function with 1*/L*.

Fixed-point iterations (FPIs) are a fundamental technique where successive iterations are computed according to (10).

(10) $$x_{k+1}\;:=g(x_{k})$$

where *g* : ℝ^*n*^ → ℝ^*n*^ is a given function. The contractive mapping theorem ensures convergence to the unique fixed point of *g* provided *g* is a contractive mapping (discussed in detail in the following section).

To optimize differentiable functions *f*, the structure of the FPIs can be leveraged by expressing *g* as shown in (11).

(11) g(x)=x−Λ∇f(x)

where ∇*f* = 0 at the extrema of *f*, rendering them as the fixed points of *g*. If Λ∇*f* satisfies the contractive property, FPIs can lead to the minima or maxima of *f*.

The Newton method exemplifies this by substituting Λ with the Jacobian of *f* at *x_k_*. Similarly, the Gradient Descent algorithm is employed, as shown in (12).

(12) Λ=η

where *η* is a predefined learning rate set by the user before the commencement, which requires careful selection to avoid divergence.

In signal and image processing, the ISTA has proven to be an effective tool for addressing sparse optimization problems. ISTA iteratively applies soft-thresholding steps to optimize the loss function and is commonly used for image reconstruction and signal compression. A specific loss function must be defined to address an optimization problem with ISTA, and appropriate tuning parameters must be set. The general formula for ISTA can be expressed as shown in (13).

(13) $$\text{minimize}\;{\left\|\mathrm{Ax}-y\right\|}_{2}^{2}+\lambda {\left\|x\right\|}_{1}$$

where *A* ∈ ℝ^*n×m*^, *m* ≫ *n* (i.e., a fat or over-complete dictionary), *y* ∈ ℝ^*n*^ is the signal, and *x* ∈ ℝ^*m*^ is the sparse code to be determined, respectively. The proximal gradient method can be used to obtain the update step to label the quadratic term as *f* and the relaxed sparsity term as *g* shown in (14).

(14) $$x^{k+1}:=S_{\lambda \eta }(x^{k}-\eta A^{T}(Ax^{k}-y))$$

where *S*_λ_ is the proximal operator for the L1-norm and soft-thresholding with parameter *λ*, as previously derived. The update step for ISTA is expressed in (15).

(15) $$x^{k+1}:=S_{\lambda /L}\left(x^{k}-\frac{1}{L}A^{T}(Ax^{k}-y)\right)$$

where *L* = *σ*_max_(*A*)^2^, representing the square of the maximum singular value of *A*, which is essentially the largest eigenvalue of *A^T^ A*. This acts as a Lipschitz constant for ∇*f* and ∇*g*. This version of the ISTA uses a fixed maximum step size to ensure the contraction of ∇*f* and ∇*g*.

FISTA is an efficient optimization method widely used in non-smooth optimization problems, particularly in sparse coding and *ℓ*_1_-regularized regression. This method combines the accuracy of gradient optimization algorithms with the efficiency of shrinkage thresholding techniques, while leveraging acceleration techniques for faster convergence. The updated formulas for FISTA are shown in (16) and (17).

(16) $$t^{k+1}=\frac{1+\sqrt{1+4{\left(t^{k}\right)}^{2}}}{2}$$

(17) $$y^{k+1}=x^{k+1}+\frac{t^{k}-1}{t^{k+1}}(x^{k+1}-x^{k})$$

where *t^k^* is the acceleration parameter used to accelerate convergence. FISTA provides a flexible and robust approach for addressing the image reconstruction problem in EIT, particularly in scenarios involving large datasets and complex data models. By integrating gradient optimization algorithms with thresholding techniques, FISTA optimizes the image reconstruction process and mitigates the effects of noise and measurement errors.

The Newton-Raphson method is an iterative approach used for unconstrained minimization when solving nonlinear functions [35, 36]. The core concept of this method involves approximating the objective function with a quadratic function and finding the minimum of this approximation, which serves as an estimate of the minimum of the original objective function. The iterative formula for this method is expressed as shown in (18).

(18) $$x^{\left(k+1\right)}=x^{\left(k\right)}-{\left(A^{T}A+\mathrm{rl}\right)}^{-1}A^{T}(Ax^{\left(k\right)}-y)$$

where *r* represents the regularization parameter, and *I* is the identity matrix. In this study, the value of *r* was set to 0.1.

The NOSER method is a fast, static EIT algorithm derived from the Newton method. However, it operates without the need for iterations [35, 34]. Instead, it only uses the first step of the Newton method. This approach can be summarized as shown in (19).

(19) $$\left\{\begin{array}{l} E(x^{\left(0\right)})={\left\|y-{\mathrm{Ax}}^{\left(0\right)}\right\|}^{2},\\ F(x^{\left(0\right)})=\frac{\partial E(x^{\left(0\right)})}{\partial x_{i}},\;\;\;i=1,2,\ldots ,N,\\ x=x^{\left(0\right)}-{\left[A(x^{\left(0\right)})\right]}^{-1}\times F(x^{\left(0\right)}), \end{array}\right.$$

### Metrics

To evaluate the efficacy of the iterative methods in enhancing the quality and accuracy of signal or image reconstruction, the following metrics were employed: Mean Absolute Error (MAE), Mean Squared Error (MSE), Signal-to-Noise Ratio (SNR), and Peak Signal-to-Noise Ratio (PSNR) [46].

The MAE metric quantifies the average magnitude of errors between the reconstructed (or predicted) values and the true values, providing a straightforward measure of reconstruction accuracy, as shown in (20).

(20) $$\text{MAE}=\frac{1}{N}\sum_{i=1}^{N}\left|Y_{i}-{\hat{Y}}_{i}\right|$$

where *Y_i_* represents the actual values, *Ŷ_i_* denotes the values predicted by the model, and *N* denotes the total number of observations.

MSE measures the average squared difference between the estimated and actual values, highlighting the variance of the reconstruction errors, as shown in (21).

(21) $$\text{MSE}=\frac{1}{N}\sum_{i=1}^{N}{\left(Y_{i}-{\hat{Y}}_{i}\right)}^{2}$$

The SNR evaluates the quality of the reconstructed signal by comparing its power with the power of the noise present, providing an indicator of the clarity of the signal as shown in (22).

(22) $$\text{SNR}=10\cdot \log_{10}\left(\frac{\sigma_{\mathrm{signal}}^{2}}{\sigma_{\mathrm{noise}}^{2}}\right)$$

where $\sigma_{\mathrm{signal}}^{2}$ and $\sigma_{\mathrm{noise}}^{2}$ are the variances of the signal and noise, respectively.

The PSNR is relevant in image processing and assesses the quality of a reconstructed image by measuring the ratio of the maximum possible pixel value to the mean squared error between the reconstructed and original images, as shown in (23).

(23) $$\text{PSNR}=10\cdot \log_{10}\left(\frac{{\mathrm{MAX}}_{I}^{2}}{\text{MSE}}\right)$$

where *MAX_I_* represents the maximum possible intensity of the image pixel.

Assessing the quality and fidelity of reconstructed images is paramount for accurate diagnosis and imaging. Various metrics have been used to gauge different aspects of the reconstruction process, shedding light on factors such as image accuracy, resolution, and shape fidelity [47].

The Amplitude Response (AR) is a metric that quantifies the proportion between the amplitudes of pixels within an image’s target area and those in its reconstructed counterpart. It is relevant when evaluating the reconstruction of a spherical target occupying a volume *V_target_* located in the electrode plane and having a conductivity of *σ_t_* within a medium with a uniform reference conductivity of *σ_baseline_*. The formula for computing the AR is shown in (24).

(24) $$\mathrm{AR}=\frac{S_{k}[\tilde{x}]k}{V_{\text{target}}\frac{\Delta \sigma }{\sigma_{\text{baseline}}}}$$

where ∆*σ* represents the difference between the target conductivity *σ_target_* and reference conductivity *σ_baseline_*, indicating the degree of deviation in the conductivity within the target from that of its surrounding medium.

The position error (PE) gauges the accuracy of aligning the target location within a reconstructed image. This metric contrasts the actual target coordinates, *r_target_*, with the centroid of the reconstructed target area, *r_recon_*, formulated as shown in (25).

(25) $$\mathrm{PE}=r_{\mathrm{target}}-r_{\mathrm{recon}}$$

A positive SD value suggests a tendency for the reconstructed targets to shift towards the center of the imaging medium.

The resolution (RES) evaluates the dimensions of the delineated targets relative to the overall imaging field and serves as an analog to the breadth of the point spread function (PSF), as shown in (26).

(26) $$\mathrm{RES}=\frac{r_{\mathrm{reconArea}}}{A_{\mathrm{total}}}$$

where *r_reconArea_* represents the aggregate pixel count within the identified target zone, *x̂_q_*, and *A_total_* is the pixel count spanning the entire area subject to the reconstruction.

Shape distortion (SD) is a reconstruction algorithm commonly yield circular representations of centrally positioned targets. However, deviations from the expected circular shape often occur, particularly for targets near the imaging medium’s boundary. The SD metric quantifies the proportion of the reconstructed image that deviates from a circular shape with equal area as shown in (27).

(27) $$\mathrm{SD}=\frac{\sum_{k\notin C}{\left[{\hat{x}}_{q}\right]}_{k}}{\sum_{k}{\left[{\hat{x}}_{q}\right]}_{k}}$$

where *C* denotes a circle centered at the centroid of *x̂_q_* with an area equivalent to that of the target.

The Ringing (RNG) metric evaluates whether the reconstructed images exhibit areas of contrasting polarity surrounding the primary reconstructed target area. RNG quantifies the ratio of image amplitudes of opposite polarity outside the circle *C* to those within *C* as shown in (28).

(28) $$\mathrm{RNG}=\frac{\sum_{i\notin C}X_{\&}{\left[\hat{x}\right]}_{i}<0{\left[\hat{x}\right]}_{i}}{\sum_{i\in C}{\left[\hat{x}\right]}_{i}}$$

## Experiments and Results

### Experiments in Signal Reconstruction

**Figure 1:**
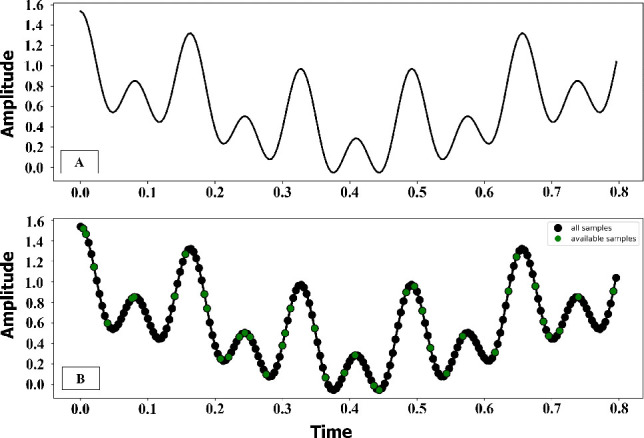
Simulate the wave signals from three frequencies of 10, 50, and 100 kHz with (A) the sum of the signal and (B) the discrete point in the signal with a threshold of 0.2.

In this study, simulation data were generated to evaluate the initial performance of the iterative algorithms. Data were generated from three sine waves with frequencies of 10, 50, and 100 Hz, amplitude of 1 V, number of samples *N_sample_* of 200, and added noise of 12 dB with subsampling locations of 0.2, as shown in Fig. 1.

**Figure 2:**
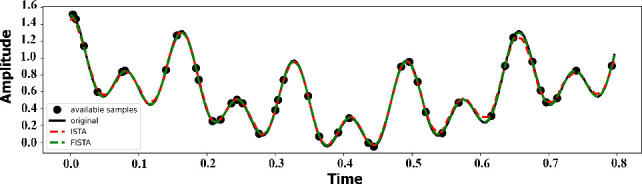
Reconstructed signal from ISTA and FISTA iterative method.

The results were compared between the two iterative methods, ISTA and FISTA, in terms of their performance in signal reconstruction for EIT applications. The results revealed that the FISTA method (R^2^ = 0.9978) improved ISTA (R^2^ = 0.9925), particularly by reducing noise and measurement errors, as shown in Fig. 2.

**Figure 3:**
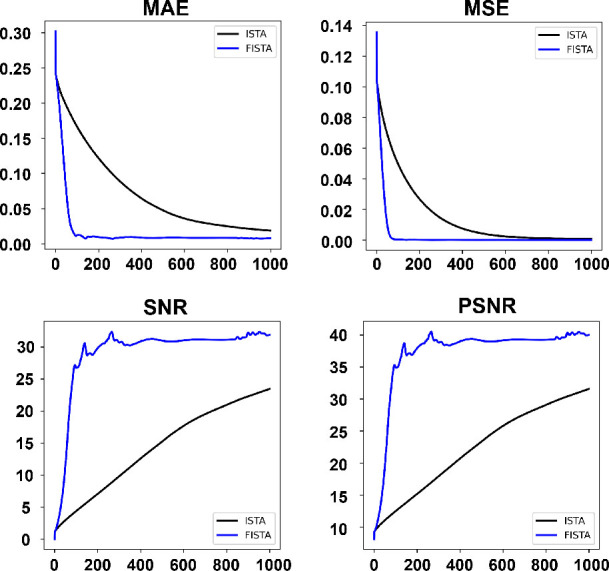
Evaluation of ISTA and FISTA performance based on MAE, MSE, SNR, and PSNR metrics over iterations.

The further analysis considered additional evaluation metrics such as the MSE, SNR, and PSNR. The results revealed that FISTA exhibited superior convergence and stability compared to ISTA, particularly between iterations of 80 and 100. Furthermore, the stable values of MSE, SNR, and PSNR for FISTA were lower than those for ISTA, with values of 0.0015, 30, and 40, respectively, compared to 0.002, 25, and 30, as shown in Fig. 3. This highlights the enhanced signal reconstruction capabilities of FISTA over ISTA. The adaptability and efficiency of FISTA stem from the integration of thresholding and gradient optimization techniques. Through this amalgamation, FISTA improves signal reconstruction and alleviates the impact of noise and measurement errors in the data.

### Experiment with 2-dimensional EIT Model Simulation

**Figure 4:**
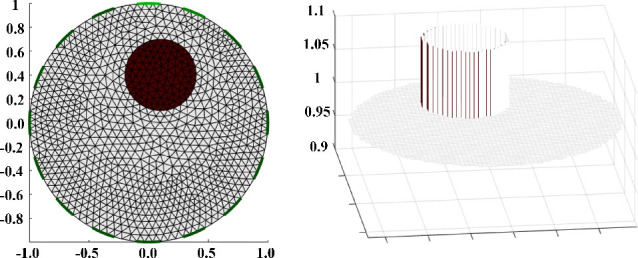
16-electrode model simulates an abnormal object with a radius of 0.3 in the phantom mesh (left) and intensity conductivity (right).

In the application of EIT, simulation data were performed using the EIDORS library and MATLAB software, with a phantom with 16 electrodes, and a circular object assumed to be the conductor was placed into the phantom with the FEM model, as shown in Figure 4 [48, 49].

**Figure 5:**
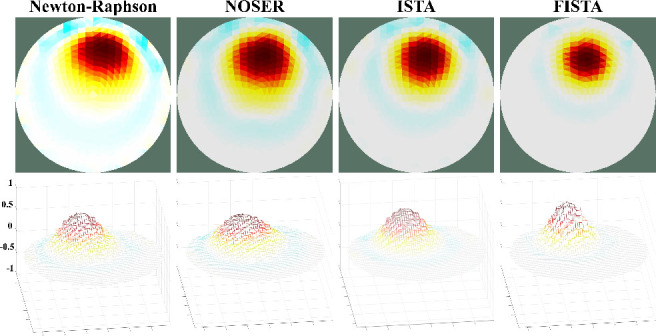
Reconstructed EIT image (top) and conductivity intensity (bottom) using Newton-Raphson, NOSER, ISTA, and FISTA regularization methods, respectively

The influence of regularization on the reconstruction of conductivity images is commonly addressed as an inverse problem. The results of the experiments with simulated EIT data are shown in Figure 5, using two iterative methods from previous studies—Newton-Raphson and NOSER—alongside the two methods investigated in this study, ISTA, and FISTA.

The results demonstrated that all methods successfully identified the presence of objects within the phantom. However, the FISTA regularization approach shows the highest convergence and highest contrast in conductivity between the object and background, followed by ISTA, Newton-Raphson, and NOSER. This comparative analysis highlights the significance of selecting an appropriate iterative method for enhancing the image quality in EIT. The performance of FISTA can be attributed to its advanced algorithmic structure that efficiently balances data fidelity and regularization, leading to more accurate and visually discernible reconstructions. Furthermore, the ability of the FISTA method to rapidly converge while maintaining a high contrast underscores its potential in applications requiring precise imaging, such as medical diagnostics and industrial process monitoring.

**Figure 6:**
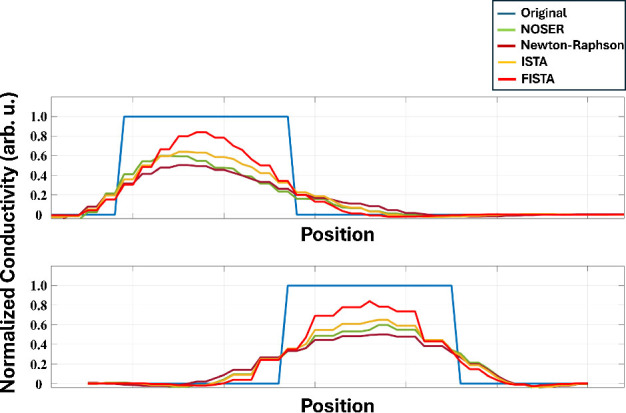
Normalized conductivity profile through the center of the object vertically (top) and horizontally (bottom).

Figure 6 shows the normalized conductivity profiles according to the vertical and horizontal axes that intersect the center of the object in Figure 5 for the four iterative methods. ISTA and FISTA show high contrasts and sensitivities at the interfaces between the object and the background. Furthermore, the FISTA reconstructs conductivity with an accuracy of over 0.8. Similarly, ISTA achieves an accuracy of more than 0.6, and Newton-Raphson and NOSER achieve an accuracy of over 0.4.

### Experiment with 3-dimensional EIT Model Simulation

**Figure 7:**
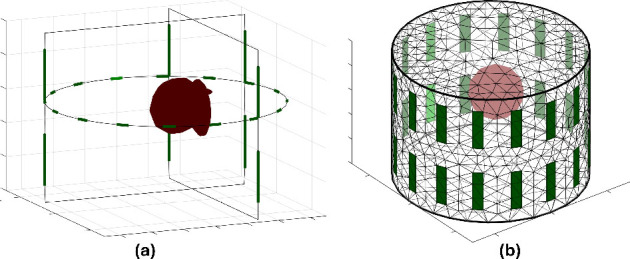
3D simulation model 32 electrodes (2 rings) of an irregular spherical object with radius 0.3 in phantom with (a) in three axes and (b) in three-dimensional space.

In the experiment with the 3D EIT model, the 32 electrodes were divided into two rings. A spherical object with a radius of 0.3 is established with conductivity twice that of the background. The hyperparameter (*α*) was set to 1e–3 for regularization. The adjacent current injection strategy was used with a current of 2.0 mA, and 512 voltage measurements. Figure 7 shows the simulated 3D model with sand slices along three axes, as shown in Fig. 7(a), and the 3D mesh observations as shown in Fig. 7(b).

**Figure 8:**
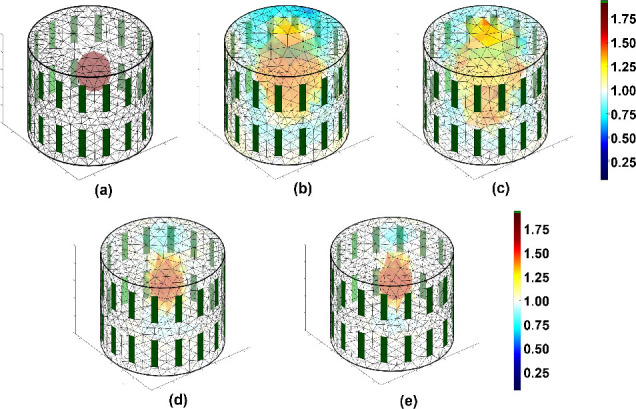
3D EIT reconstruction using iterative methods with (a) original object, (b) Newton-Raphson, (c) NOSER, (d) ISTA, and (e) FISTA with corresponding conductivity scale.

Figure 8 shows the reconstruction of the 3D image using different iterative methods from the original structure shown in Fig. 8(a). The internal structure, which was difficult to observe in Fig. 8(b) when using Newton-Raphson, and Fig. 8(c), when using NOSER, becomes more obvious with ISTA. Fig. 8(d) and FISTA Fig. 8(e), respectively.

**Figure 9:**
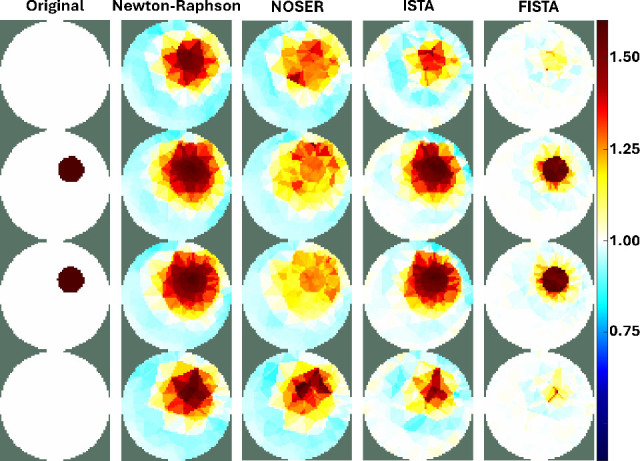
Cross-section of the 3D EIT image shown in Fig. 8 corresponds to the iterative methods from left-to-right original object, Newton-Raphson, NOSER, ISTA, and FISTA, respectively.

The effectiveness of the proposed technique was validated through cross-sections of the 3D structure, with the original structure shown in Fig. 9(a). The blur effect is better suppressed by the iterative methods FISTA and ISTA than by the Newton-Raphson and NOSER methods, as shown in Figs. 9(b)–(e), respectively.

### Experiments in Heterogeneous Tissue Environments

**Figure 10:**
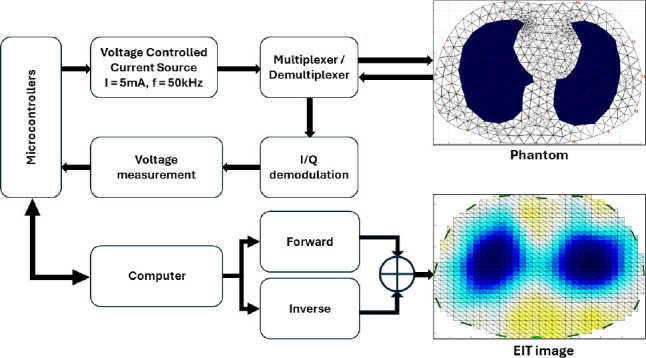
Process of signal acquisition and image reconstruction of the EIT device.

**Figure 11:**
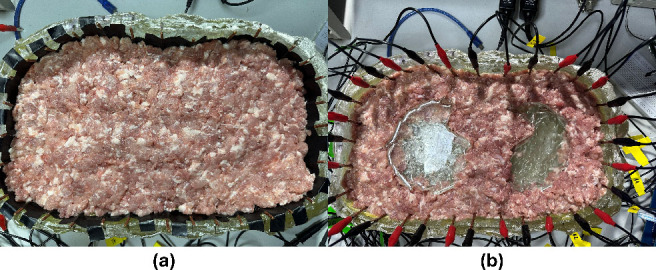
Phantom model with ground pork meat and acrylic resin objects simulating the lung shape with (a) without objects and (b) with objects.

**Figure 12:**
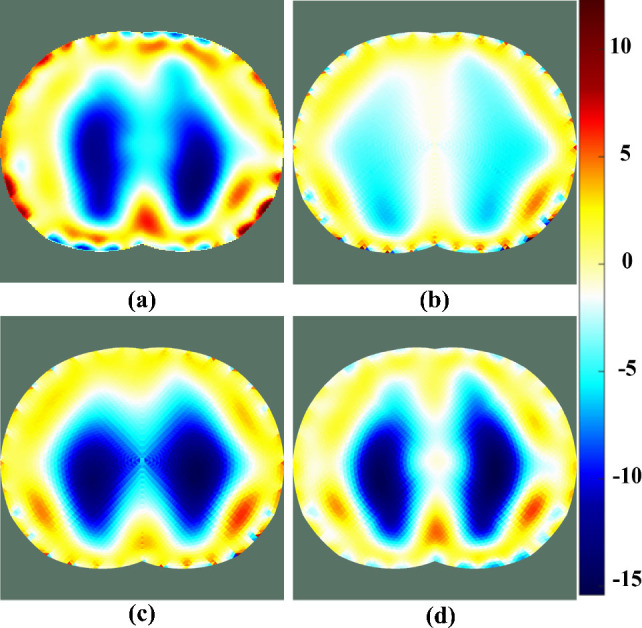
EIT image reconstructed from the lung simulation phantom using iterative methods with (a) Newton-Raphson, (b) NOSER, (c) ISTA, and (d) FISTA.

Validation experiments with a heterogeneous tissue environment. The experiment used a background environment of ground meat filled with phantom material. The ground pork meat used was commercially prepared and purchased from a supermarket, ensuring compliance with ethical standards. Two objects made of acrylic resin were placed, with shapes that mimic the shape of the left and right lungs. A total of 32 electrodes were used with the adjacent current injection strategy (I = 5.0 mA) at a frequency of 50 kHz, and 1024 voltage values were acquired. The 2D FEM model is set up with a high vertex density and is two-dimensional. The tuning hyperparameter was set to 1e–3 and the time-difference rendering method was used. The impedance-signal acquisition device used in this study is shown in Fig. 10. Fig. 11 shows an experimental phantom without an object, as shown in Fig. 11(a) and with the object, as shown in Fig. 11(b):

Figure 12 shows a reconstructed EIT image of the experimental phantom. The blurred and unrecognizable edges of the lung simulation object when using Newton-Raphson in Fig. 12(a), and NOSER in Fig. 12(b) are improved by using ISTA in Fig. 12(c), and FISTA in Fig. 12(d). In addition, FISTA exhibits high convergence. Therefore, the shape of the reconstructed object closely approximated its actual shape.

### Ethical approval

The conducted research is not related to either human or animal use.

## Discussion

**Figure 13:**
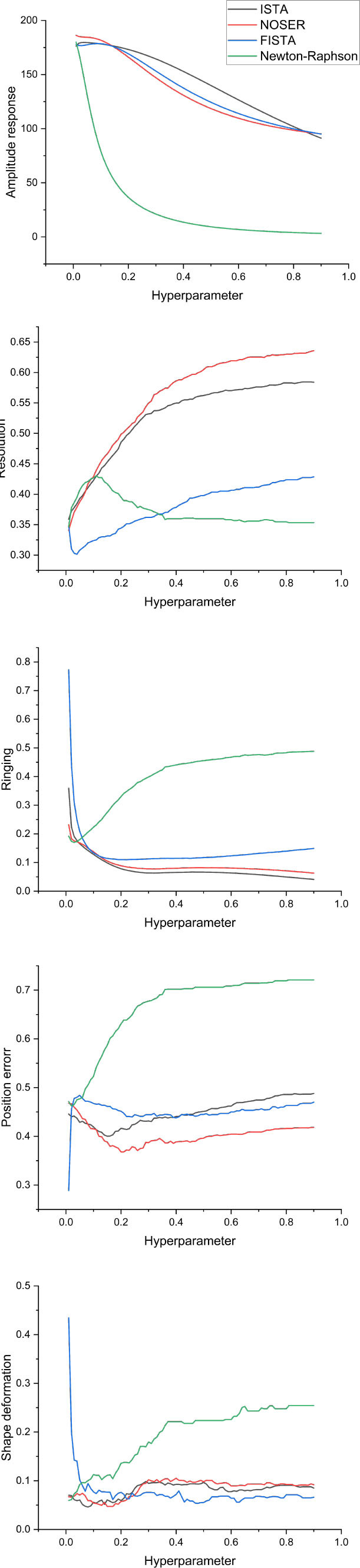
EIT image evaluation parameters reconstructed when tuning hyperparameters.

Figure 13 shows the variation in evaluation metrics for EIT images across four iterative methods within a hyperparameter range of 0 to 0.9. All methods exhibit stability within the range of 0 to 0.1 for the AR index, except for Newton-Raphson, which experiences significant fluctuations. As the hyperparameter increases from 0.1 to 0.9, the AR index for NOSER, ISTA, and FISTA decreases gradually, whereas the index for Newton-Raphson approaches zero.

For the PE index, Newton-Raphson performed the worst across the explored hyperparameter range. ISTA demonstrated the lowest PE index from 0.1 to 0.9, while FISTA and NOSER show minimal differences throughout this range. Regarding resolution, notable discrepancies are observed among the methods. Below 0.3, FISTA achieves the lowest index, indicating the best resolution, while NOSER exhibits the highest index, followed by ISTA. Beyond this range, Newton-Raphson lags significantly behind NOSER until approximately 0.3, registering an index of 0.35.

For the SD index, all methods except for Newton-Raphson exhibited optimal indices below approximately 0.1, with minimal disparities among them. However, Newton-Raphson demonstrates a high SD index, indicating less effective performance. Regarding the ringing artifact, Newton-Raphson shows the highest index across the surveyed range, indicating lower effectiveness in suppressing this artifact. In contrast, the other three methods, NOSER, FISTA, and ISTA, maintained low ringing indices, ranging from approximately 0.2 to 0.9. These three methods exhibited similar performance and demonstrated relatively good effectiveness in reconstructing EIT images. In comparison, the Newton-Raphson method showed a larger deviation from the other methods, highlighting its relative inefficacy.

The investigation into the effects of hyperparameters on the performance of the iterative methods was conducted within a specific, limited range. This constrained exploration may not encompass the optimal settings for all scenarios, potentially affecting the generalizability of the findings. A more comprehensive exploration of the hyperparameter space could potentially reveal further improvements in the reconstruction quality.

## Conclusion

The results of this study demonstrate the efficacy of ISTA and FISTA iterative algorithms in improving the accuracy and reliability of EIT image reconstruction. The evaluation of these iterative algorithms underscores their effectiveness in mitigating the effects of noise and measurement errors, thereby enhancing the quality of the reconstructed images.

One key observation is the advantage of FISTA over ISTA in signal reconstruction for EIT applications. FISTA exhibited faster convergence and greater stability, resulting in lower MSE values and higher SNR and PSNR values than ISTA. Specifically, FISTA achieved an MSE of 0.0015, an SNR of 30, and a PSNR of 40, whereas ISTA achieved an MSE of 0.002, an SNR of 25, and a PSNR of 30. These results suggest that FISTA not only optimizes image reconstruction process but also minimizes noise and measurement errors, making it a preferred choice for applications requiring high-quality imaging, such as medical diagnostics and industrial process monitoring.

Moreover, a comparative analysis of other iterative methods, including Newton-Raphson and NOSER, highlights the importance of selecting the appropriate iterative technique to improve the image quality in EIT. While all methods successfully identified objects within the phantom, FISTA exhibited the highest contrast in conductivity contrast between the object and the background, indicating its superior performance in accurately delineating targets.

The normalized conductivity profiles along the vertical and horizontal axes further confirm the effectiveness of FISTA in reconstructing the conductivity with high accuracy and sensitivity at the interfaces between the object and the background. This finding reinforces the potential of FISTA as a robust iterative method for enhancing the reliability of EIT image reconstruction across various applications.
